# Usability testing of an electronic patient-reported outcome system linked to an electronic chemotherapy prescribing and patient management system for patients with cancer

**DOI:** 10.1016/j.heliyon.2023.e16453

**Published:** 2023-05-23

**Authors:** Christel McMullan, Sarah E. Hughes, Olalekan Lee Aiyegbusi, Melanie Calvert

**Affiliations:** a Institute of Applied Health Research, University of Birmingham, Birmingham, UK; b Centre for Patient Reported Outcomes Research, University of Birmingham, Birmingham, UK; c NIHR Surgical Reconstruction and Microbiology Research Centre, University of Birmingham, Birmingham, UK; d NIHR Birmingham Biomedical Research Centre, University of Birmingham, Birmingham, UK; e NIHR Birmingham-Oxford Blood and Transplant Research Unit (BTRU) in Precision Transplant and Cellular Therapeutics, University of Birmingham, Birmingham, UK; f National Institute for Health and Care Research (NIHR) Applied Research Collaboration West Midlands, Birmingham, UK; g Birmingham Health Partners Centre for Regulatory Science and Innovation, University of Birmingham, Birmingham, UK

**Keywords:** Usability study, Electronic patient reported outcomes, Cancer, Think aloud

## Abstract

**Background:**

People affected by cancer experience a wide range of symptoms which have a major impact on their functioning and health-related quality of life (HRQoL). One way to measure the impact of cancer symptoms is through the use of patient-reported outcomes.

**Methods:**

An electronic patient-reported outcome (ePRO) application (ChemoPRO®) was designed to be used by cancer patients to report their symptoms and communicate with their clinical team. Usability testing sessions were conducted with people with lived experience of cancer to understand how real users interact with the ChemoPRO® system. One-to-one testing sessions were conducted to assess use of the system and identify areas for further refinement. User satisfaction was assessed using a brief satisfaction questionnaire previously used by Aiyegbusi et al. (date).

**Results:**

Ten people with lived experience of cancer took part in the usability study. Symptoms and HRQoL measures, including the Euroqol EQ5D5L and the PRO-CTCAE™ were included in the ePRO system.

**Participants:**

had a mean age of 62.3 years. Three critical errors and 21 non-critical errors were reported. All participants were enthusiastic about the app. Participants liked the simplicity and responsiveness of the patient-facing app and highlighted the potential for communicating with their clinical team. The overall usability and satisfaction score was 4.5 (sd = 0.09).

**Conclusion:**

This usability study suggests that people with lived experience of cancer found the ChemoPRO® app acceptable and easy to use. One of the key features of this particular ePRO system that should be developed further is system functionality to facilitate communication between patients and clinicians. Future testing should include testing in a clinical setting and testing with people from ethnic minorities.

## 1 Introduction

In 2020, there were around 20 million new cancer cases worldwide and it is estimated that this number will rise to over 27 million by 2040 [1]. Similarly, cancer incidence has increased in the United Kingdom (UK) by 19% in the last decade and will continue to do so [2]. Each year, there are around 450,000 new cancer cases (605 per 100,000) [3]. It is estimated that, in the UK, around three million people are currently living with cancer [4]. These patients may experience a wide range of disease and treatment symptoms and treatment-related toxicities that greatly affect their functioning, heath-related quality of life (HRQoL) and their impact on healthcare resource use [5,6]. Patients’ journeys through cancer treatment are increasingly captured with electronic systems, such as ChemoCare® which is in use in several National Health Service (NHS) trusts in the UK [7].

ChemoCare® is an electronic chemotherapy prescribing and patient management system. The electronic platform was designed by CIS Oncology (https://www.cis-healthcare.com/), an ISO certified med tech company based in the UK (ISO 13485:2016, EN ISO 13485:2016, ISO 9001:2015, ISO 27001:2013). ChemoCare® is used for prescribing, preparing and administering chemotherapy, as well as scheduling treatment and sharing data. It allows instant access to data by the clinical team and patients, ensuring accurate and fully informed prescribing at all times. However, to date, the system does not include any patient reported outcomes (PROs), measures of health status coming directly from the patient without interpretation by a clinician or anyone else [8]. Clinical teams and patients are keen to see PROs included in the electronic platform to assess the impact of disease and treatment on patients’ symptoms and quality of life from a patient perspective. Electronic capture of PROs (ePROs), using online validated questionnaires, has important advantages over paper forms [[9], [10], [11]]. The collection of ePROs removes the need for manual data entry which carries the risk of human error and facilitates prompt review by the clinical team to tailor care to individual patient needs [10,11].

PRO assessment in an oncology setting offers a range of potential benefits for individual patient care and for clinicians, regulators, healthcare management teams, commissioners, and policy makers. Benefits include improved symptom management and symptom control and enhanced patient-clinician communication, patient satisfaction and well-being [9,12]. In addition, in the US the use of PROs to monitor patient symptoms in routine cancer care has been shown to lead to clinical benefits include fewer A&E visits, hospitalisations and an increase in survival [9].

## 2 ChemoPRO®

ChemoPRO® is an app linked to the ChemoCare® platform described above. It was designed by CIS Oncology, with ongoing input from clinical stakeholders. The ChemoPRO® app helps hospitals and other stakeholders maintain contact with their patients in between visits. Patients can record symptoms and wellness which can be accessed by the healthcare team. It also allows patients to access a range of information, such as appointments and medication reminders.

The ChemoPRO® app consists of several modules: ‘Appointments’, ‘How are you?‘, ‘My symptoms’, ‘Logs’, ‘Info’, and ‘Help’. The ‘How are you?’ and ‘My symptoms’ modules refer to two PROMs (EQ5D-5L and PRO-CTCAE™, as outlined in methods), as shown in Picture 1.

CIS Oncology are GDPR and ISO 27001 compliant to ensure that all patient data is held securely in line with current best practice and legislation.

### 2.1 Usability testing

**Fig. 1:**
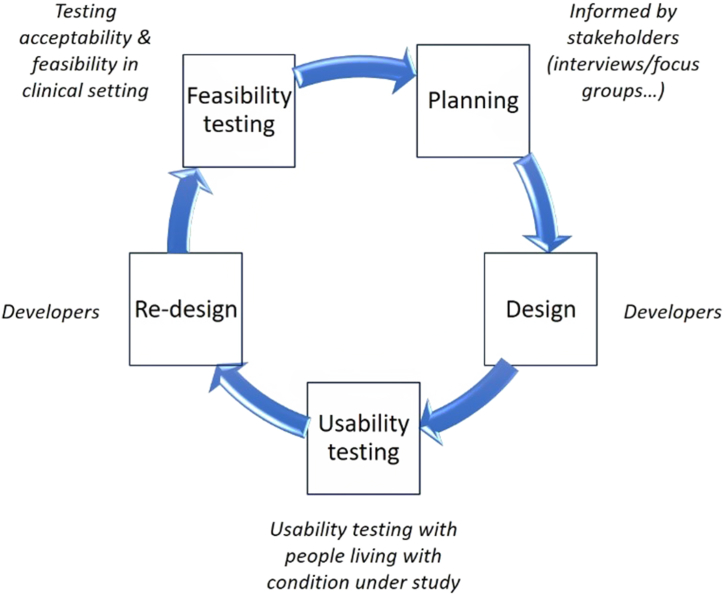
Stages of system/product development.

It is vital that the user-friendliness and usability of ePRO systems are assessed and improved prior to implementation in a clinical setting to reduce attrition rates and to ensure that the system performs efficiently and effectively [13]. Usability testing is one of the stages of system development (Fig. 1).

Usability testing is a technique that involves the testing of systems, products or websites with participants drawn from the target population. It aims to assess how effective and efficient systems are, as well as users’ satisfaction with the system [14]. It can be conducted during a single session or in a more iterative manner during several sessions [15]. In our case, it allows end users to actually test ePRO systems and can assist ePRO developers in the evaluation of ePRO user interface [15].

Therefore, the aim of this study was to assess the usability of the CIS Oncology ChemoPRO® platform with individuals with lived experience of cancer.

## 3 Methods

### 3.1 Ethical considerations

This usability study was approved by the University of Birmingham Research Ethics committee (ERN_22-0510).

### 3.2 Study participants and recruitment

People with lived experience of any type of cancer were recruited by sharing the study advert on social media (Twitter) and with the National Cancer Research Institute (NCRI) Consumer Forum and a Patient and Public Involvement (PPI) panel based in Birmingham in November 2022. Further inclusion criteria can be found in Box 1.

Box 1 – list of inclusion criteria

**Figure:**
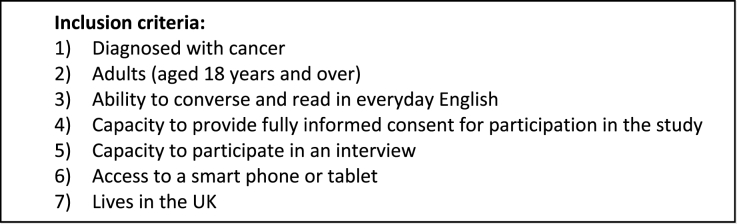

**Fig. 2:**
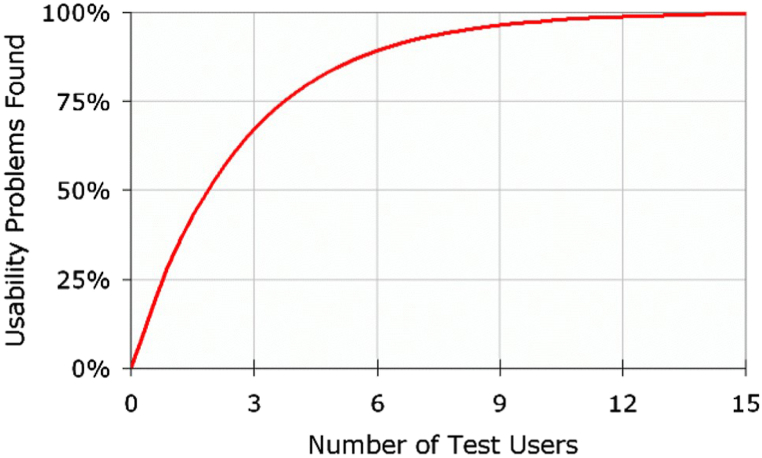
Sample sizes for usability studies (reproduced with permission of the Nielsen Group) [19].

Previous research informed our sample size [[15], [16], [17], [18], [19]]. It has been shown that 8-10 participants, and sometimes as low as 5 if planning to do a series of testing sessions [18], are required to detect over 80% of issues [17,19], as shown in Fig. 2.

By the time we interviewed the last participant, it was apparent that we had reached saturation, as no new issue was being mentioned.

People interested in taking part in the study contacted the researcher (CM) who emailed them to confirm their eligibility and sent them a participant information sheet and a consent form to consent to participation in the study and publication of the results. A mutually suitable date and time to conduct the usability testing session was then arranged, after obtaining written informed consent. In total, nine people with cancer were recruited.

People who completed the study were given a £10 voucher in return for their participation in the study.

Clinicians were not included in the current usability study as they have been heavily involved in the planning and design of the app.

### 3.3 ChemoPRO® registration process

**Fig. 3:**
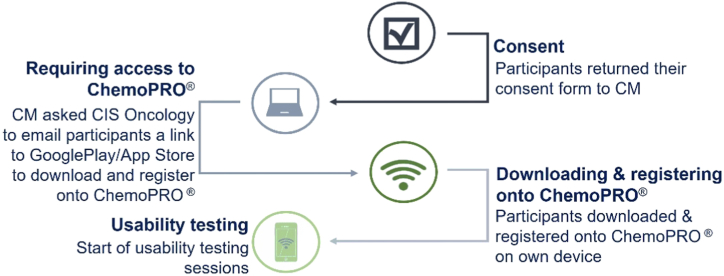
Process to consent, download and register onto ChemoPRO®.

Once participants gave their informed consent, CIS Oncology emailed them a link to download and register onto the ChemoPRO® app. This invitation email included a link to GooglePlay/App Store and a numerical code to enter after downloading the app. Participants then downloaded the app and registered (Fig. 3).

**Picture 1:**
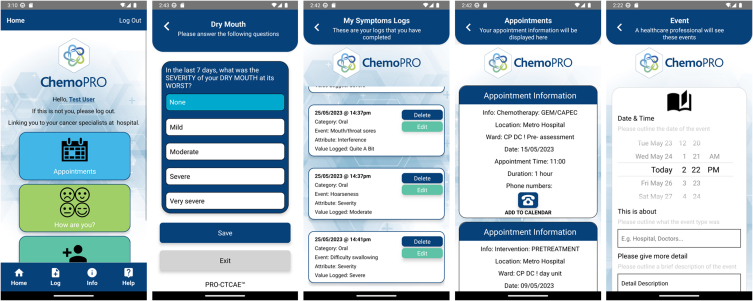
Screenshots of the ChemoPRO® app.

### 3.4 PROs

#### 3.4.1 EQ-5d-5L

The Euroqol EQ-5D-5L [20] comprises 5 items plus 1 visual analogue scale. It has been widely validated across a range of conditions and used to assess health outcome from a wide variety of interventions on a common scale, for purposes of evaluation, allocation and monitoring [21,22]. It is used by the National Institute for Health and Care Excellence (NICE) in health technology assessment. EQ-5D-5L, takes only a few minutes to complete and has a same day recall period.

#### 3.4.2 PRO-CTCAE™

The Common Terminology Criteria for Adverse Events (CTCAE) is routinely used in oncology as the means of categorising adverse events in trials and is a descriptive terminology that evolved alongside other toxicity grading systems to become a comprehensive, multi-modality grading system for reporting the acute and late effects of cancer treatment [21,22]. The PRO-CTCAE™ was developed to characterise the frequency, severity and interference of 78 symptomatic treatment toxicities that could be meaningfully reported from the patient perspective. It has been established that symptomatic adverse events are under-detected by clinicians versus patients, who are better at identifying low-grade symptomatic events [23,24]. As such, the PRO-CTCAE™ could generate information that complements CTCAE data and can provide a more holistic insight into patient experience on treatment, which is integral for clinical decision-making. In oncology, the PRO-CTCAE™ has demonstrated favourable validity, reliability, and responsiveness [25].

### 3.5 Testing sessions

The one-to-one testing sessions consisted of performing a series of tasks on the app whilst talking to the researcher about their experience (‘Think Aloud’ technique) [26]. The list of tasks performed by the participants can be found in Box 2. Although one of these tasks was to complete the two PROMs included, we did not use the responses to the questionnaires to assess the usability of the app. This will be part of the next stage of the evaluation, the feasibility study. Effectiveness was assessed by recording the type and number of errors. Critical errors were errors that prevented participants from using the app and completing the tasks unless the researcher stepped in and advised participants on how to resolve the issue. Conversely, non-critical errors were minor errors that did not prevent participants from completing the testing session. Efficiency was assessed by providing an estimation of the time it took participants to complete the EQ5D5L PROM. Participants also identified areas of improvement and made suggestions to improve the app and the users' experience. This was followed by a short qualitative interview to capture their overall experience of using the app (retrospective probing) [27]. Finally, participants were asked to complete a short demographics questionnaire and a brief satisfaction questionnaire previously used by Aiyegbusi et al. [16] The satisfaction questionnaire included four questions to rate the usability of the system and participants' satisfaction on a 5-point scale (1 = very dissatisfied, 5 = very satisfied) [16].

Box 2 – List of tasks performed by participants.

**Figure:**
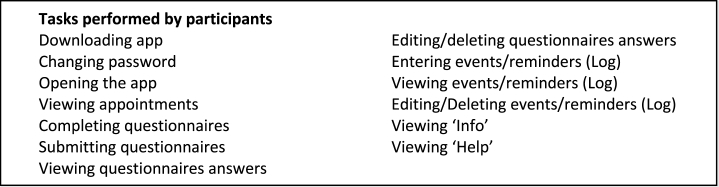

### 3.6 Data analysis

Participant characteristics were summarised as n (%). Comments from all the participants were recorded in a table alongside observations from the researcher, whether participants completed the tasks independently or with support, how they found the user experience, and what could be done to improve the ChemoPRO® app. Participants’ comments during the interviews were extracted as quotes and categorised under “Overall comments and impressions” (Table 5). Participant ratings for the four usability questions were used to calculate a mean score per question.

## 4 Results

### 4.1 Participant characteristics

Ten people with lived experience of cancer took part in this usability study. This included six women and four men. The mean age of the participants was 62.3 years (range = 44–76 years). Table 1 presents the participants’ characteristics.

**Table 1:** Participant characteristics (n = 10).

Characteristics	n (%)
Age (years)	
41–50	2 (20)
51–60	2 (20)
61–70	3 (30)
71–80	3 (30)
Gender	
Female	6 (60)
Male	4 (40)
Ethnicity	
White	10 (100)
Employment status	
Employed	6 (60)
Retired	4 (40)
Education level	
A-Levels or equivalent	2 (20)
Degree or equivalent	4 (40)
Masters/PhD or equivalent	4 (40)
Cancer diagnosis	
Leukaemia	2 (20)
Breast	3 (30)
Multiple myeloma	1 (10)
Stomach	1 (10)
Bowel	1 (10)
Rectal	1 (10)
Sarcoma	1 (10)
Level of confidence with technology	
Confident using technology	8 (80)
Not confident using technology	2 (20)
Devices used	
Android smartphone	5 (50)
iPhone	4 (40)
iPad	1 (10)

### 4.2 Assessment of effectiveness

There were three critical errors and 21 non-critical errors (Table 2). The critical errors related to the email invitation to download the app which were either not received by participants (n = 2) or were illegible (n = 1). In the case of the illegible email, the researcher advised the participant to download the app directly from GooglePlay/the App store and use the login code which was in the invitation email (and easily readable). The researcher became aware that two participants who did not receive the invitation email when she checked with the participants the day before the interview. CM notified CIS Oncology staff, who sent another invitation email.

**Table 2:** Number of errors experienced by participants.

	Participants confident with technology (n = 8)	Participants not confident with technology (n = 2)
Critical errors	2	1
Non-critical errors	16	5

The 21 non-critical errors included missing information (under Appointments and About my Hospital), not knowing how save logs, and not knowing how to change password when invited to do so.

### 4.3 Assessment of efficiency

Although it was difficult to assess the time it took participants to complete each PROMs (especially PRO-CTCAE™) as they stopped to talk to the researcher during the process, it is estimated it took participants between 1 and 2 min to complete the EQ5D5L.

## 5 Suggestions for improvement

Participants identified potential areas to improve the user experience of ChemoPRO® (Table 3).

**Table 3:** Summary of improvements to ChemoPRO® suggested by participants.

Issue	Details	Suggestions made by participants
Content does not always fit on a mobile device screen	• Some words/numbers were truncated	• Update software to facilitate viewing and reading content
Lack of clarity & transparency	• Not always clear about what the app is about when first open it.	• Add an introductory page
	• Pop-up message about participants' data not being monitored by clinical team was a little confusing for them. They wondered why they were completing the PROs.	• Delete or reword pop up message
	• Give patients as much as information as possible on upcoming appointments	• Add details such as information on availability of car parks, videos of where to go in hospital/ward
	• Not always obvious that appointments with a white background are appointments already attended	• Add “Previous appointments” to appointments with white background
Communication with clinical team	• Potential for appointments section to be more interactive	• Add tick box which users would tick if unable to attend appointments, which would be seen by clinical team.
Unnecessary items	• Some pop-up messages (warning about their answers not being monitored by the clinical team) appear every time participants uses any of the app's features or after every question answered.	• Remove unnecessary items to avoid overburden
	• Terms & conditions section is too long and technical	• Remove Terms & conditions
Difficulty reading/understanding list of previous symptoms	• Provide additional ways to view previous symptoms.	• Provide line graphs to make it easier for some people to view and understand their data.
Wrong questions in ‘Your symptoms’	• Some of the questions in ‘Your symptoms’ did not match the symptom associated with it.	• Review and change the questions/symptoms

These issues included (i) the app content not always fitting on a small phone screen and the participants not being able to read everything, (ii) lack of clarity, (iii) removing unnecessary items to avoid overburdening the users while at the same time giving them enough information (about their upcoming appointments, for example), (iv) extending communication with clinical team, and (v) providing additional ways to view their previous symptoms.

### 5.1 Assessment of satisfaction and opinion of the ChemoPRO® app

Table 4 presents participants’ rating of the usability and their satisfaction with ChemoPRO®. The mean score for individual question was high and the overall average score for usability and satisfaction was 4.5 (5-point scale).

**Table 4:** Usability and satisfaction score.

Question	Average score (sd)
Ease of use and navigation	4.4 (0.51)
Satisfaction with content	4.6 (0.51)
Satisfaction with visual display	4.5 (0.52)
Likelihood of using again or recommend it to others (when available)	4.6 (0.69)
Average usability and satisfaction score	4.5 (0.09)

**Table 5:** Participants' comments and researcher's observations.

Patients' comments	Very good, easy to use, very responsive (Participant E)
	Not quite there yet but it has potential to be useful, I like having everything in one place (Participant H)
	Graphics are nicely done, tasteful, looks as it is easy to use (Participant K)
	Clear, straightforward, easy to use, I wish I had something like that. Having that link with your clinician/clinical team is good (Participant L)
	Script size right, content tracking, would be brilliant, the beauty of it's not only great for patients and it's a great way to communicate with clinicians. Neat way of recording (Participant D)
	The “Attention” message could be simpler, it sounds too techy (Participant K)
	I can only see half of 1 and half of 8 (Participant F)
Researcher's observations	Dummy data under “Appointments” was not present in app for all participants.
	Several participants seemed confused around what an “event” was.
	Most of the participants had to be shown how to save an event/reminder i.e. click on edit in order to be able to complete the task.
	It was clear that some of the participants felt tired as the testing session went on.

### 5.2 Participants overall comments and impressions of ChemoPRO® app and researcher's observations

Overall, all participants were very positive about using the ChemoPRO® app. They found the app easy to use and navigate. They liked the colour scheme, the layout and found the buttons responsive and had no issue around online safety. Participants believed ChemoPRO® had potential for improving communication with clinicians and keeping a record for patients and clinicians of their symptoms/quality of life data over time. They were particularly impressed with the Appointments section, which they thought would help them remember when and where their appointments are as well as what their appointment was about. They liked the ability to view their previous answers to the questionnaires and being able to see if their symptoms/quality of life improved over time, or not. Participants completed and submitted the questionnaires without any issue.

Patients' overall comments and impressions of the app, as well as the researchers’ observations can be found in Table 5.

## 6 Discussion

This article presents the results of the usability testing of the ChemoPRO® app used by people with lived experience of cancer. In addition to two PRO measures (EQ-5D-5L and PRO-CTCAE™), ChemoPRO® included several features designed to facilitate management of the users’ condition. Participants found the app easy to use and navigate and completed the questionnaires with ease, despite encountering a few issues. The errors found were mainly due to programming issues with the app, demonstrating the value of usability testing. Participants believed the app had potential to facilitate communication with their clinical team and suggested areas for further refinement.

This study adds to the existing body of research focusing on testing the usability of a new electronic applications. Usability testing is a key factor for successful implementation [16], however, this is a stage that is often overlooked [28]. All participants used their own “Bring Your Own Device” (BYOD). The app was used successfully across all age groups. Although two participants (both aged over 60 years old) said they did not feel very confident using technology, both completed the usability testing unaided without any major issues [16,29]. This may be due to the fact that more older people are using smartphones on a regular basis and that smartphones facilitate digital communication [30].

Although it was difficult to time how long completing the questionnaires took, as the participants stopped often to talk to the researcher, it became clear that some of the participants were starting feel ePRO fatigue (shown by lack of concentration or forgetfulness). This might cause users to disengage from using the app. Although this is an important aspect to consider, previous work has shown that cancer patients have successfully completed all items of the PRO-CTCAE™ [31].

One of the ways we recruited participants was through social media. Although recruiting participant on social media has advantages, such as being timesaving and inexpensive, it can pose some problems, such as having less control over who joins the study (in the case of passive recruitment). In our case, several people who asked to join the study (n = 6) were not included in the study as the researcher (CM) had strong doubts about their eligibility and motivation for joining the study. The email messages sent to express their interest in the study were worded exactly the same, were not signed, and contained the same grammar mistakes. The first two of these people who wanted to join the study never replied to CM's request to set a date/time for the testing session. Their motivation may have been the voucher offered to participants, which, although was not included on the study ad posted on social media, was included in the participant information sheet. As a result, a lot of time was spent to identify the potentially ‘fake’ participants, which defeats the purpose of using social media to recruit research participants.

## 7 Implications for ePROMs developers

Some participants thought that some of the features of the app would benefit from being removed in order to make the app as simple as possible and avoid overburdening users. Therefore, ePRO programmers need to ensure that its use is as simple as possible for participants.

Participants liked the fact that they were able to view their previous answers to the questionnaires. However, most of them thought that presenting previous answers in a visual way (i.e., line graph) rather than in a list form (as it currently is the case) would help them understand how their symptoms/quality of life have changed overtime. It is recommended that developers follow PROTEUS Consortium's existing guidance on presentation of PRO data [32,33].

## 8 Strengths and limitations

The main strength of our study is its methodology which includes a ‘think aloud’ technique which provides real-time feedback.

We are aware that our study has limitations. The sample size limited the diversity within our participant sample. Although we actively tried to recruit a diverse sample and participants in our usability study were all highly educated. Despite reaching out to a number of PPI groups and charities, including charities working specifically with ethnic minority groups, three people from ethnic minorities declined taking part in our study. One explanation for having difficulties finding participants from ethnic minorities is the lower cancer incident rate in the Asian and Black ethnic groups, and in people of mixed or multiple ethnicity, compared with the White ethnic group, in England [2]. This will be addressed in the next stage of the study which will be a feasibility study. The feasibility study will also include a larger sample and will test the electronic platform in a clinical setting on a long-term basis.

It was difficult to assess efficiency as participants stopped to talk to the researcher while completing the questionnaires, especially PRO-CTCAE™. Although it was not possible to provide an estimate of time it took participants to complete the process, prior work with PRO-CTCAE™ suggest that on average it takes users 11.1 s to answer one question (sd = 8.4) [34].

## 9 Conclusion

Our study showed people with lived experienced of cancer can easily report their symptoms/quality of life through the use of an electronic system. One of the key features of this particular ePRO system that should be developed further is the app's ability to facilitate patient-clinician communication. Future testing should include testing in a clinical setting and testing with people from ethnic minorities and people with a lower education level.

## Author contribution statement

Christel McMullan: Conceived and designed the experiments; Performed the experiments; Analyzed and interpreted the data; Contributed reagents, materials, analysis tools or data; Wrote the paper. Sarah E Hughes; Olalekan Lee Aiyegbusi; Melanie Calvert: Conceived and designed the experiments; Analyzed and interpreted the data; Wrote the paper.

## Funding statement

This study was funded by CIS Oncology.

## Ethics statement

This usability study was approved by the University of Birmingham Research Ethics committee (ERN_22-0510).

## Data availability statement

Data will be made available on request.

## Declaration of competing interest

The authors declare that they have no known competing financial interests or personal relationships that could have appeared to influence the work reported in this paper.

## References

[1] Sung H. (2021). Global cancer statistics 2020: GLOBOCAN estimates of incidence and mortality worldwide for 36 cancers in 185 countries. CA A Cancer J. Clin..

[2] UK, C.R. Cancer Statistics for the UK. Available from: https://www.cancerresearchuk.org/health-professional/cancer-statistics-for-the-uk#heading-Zero.

[3] UK, C.R. Cancer incidence for all cancers combined. 06/12/22]; Available from: https://www.cancerresearchuk.org/health-professional/cancer-statistics/incidence/all-cancers-combined#heading-Zero.

[4] Support, M.C. Prevalence by cancer type, nation, sex and year. 2020; Available from: https://www.macmillan.org.uk/dfsmedia/1a6f23537f7f4519bb0cf14c45b2a629/5192-10061/macmillan-2020-cancer-prevalence-figures-and-methodology.

[5] Basch E. (2020). Clinical utility and user perceptions of a digital system for electronic patient-reported symptom monitoring during routine cancer care: findings from the PRO-TECT trial. JCO Clin. Cancer Inform..

[6] Henry D.H. (2008). Symptoms and treatment burden associated with cancer treatment: results from a cross-sectional national survey in the U.S. Support. Care Cancer.

[7] McDonald L., Stein D., Carroll R., Soni M., Schultze A., Ramagopalan S., Wagstaff J. (2019). Extracting data from a chemotherapy prescription platform for real-world oncology research in the UK: a pilot study. Future Oncol..

[8] FDA (2009). https://www.fda.gov/media/77832/download.

[9] Basch E. (2016). Symptom monitoring with patient-reported outcomes during routine cancer treatment: a randomized controlled trial. J. Clin. Oncol..

[10] Muehlhausen W. (2015). Equivalence of electronic and paper administration of patient-reported outcome measures: a systematic review and meta-analysis of studies conducted between 2007 and 2013. Health Qual. Life Outcome.

[11] McMullan C. (2020). Care providers' and patients' attitudes toward using electronic-patient reported outcomes to support patients with traumatic brain injury: a qualitative study (PRiORiTy). Brain Inj..

[12] Gilbert J.E. (2012). Quality improvement in cancer symptom assessment and control: the provincial palliative care integration project (PPCIP). J. Pain Symptom Manag..

[13] Maramba I., Chatterjee A., Newman C. (2019). Methods of usability testing in the development of eHealth applications: a scoping review. Int. J. Med. Inf..

[14] Standardization I.O.f. (2018).

[15] Aiyegbusi O.L. (2020). Key methodological considerations for usability testing of electronic patient-reported outcome (ePRO) systems. Qual. Life Res..

[16] Aiyegbusi O.L. (2018). Development and usability testing of an electronic patient-reported outcome measure (ePROM) system for patients with advanced chronic kidney disease. Comput. Biol. Med..

[17] B, B. Determining the correct number of usability test participants: usability.gov. 2006 [cited April 2023; Available from: https://www.usability.gov/get-involved/blog/2006/09/correct-number-of-test-participants.html.

[18] J, N. Why you only need to test with 5 users: Nielsen Norman Group. 2000 April 2023]; Available from: https://www.nngroup.com/articles/why-you-only-need-to-test-with-5-users/.

[19] J N. (1993).

[20] EuroQol G. (1990). EuroQol--a new facility for the measurement of health-related quality of life. Health Pol..

[21] Trotti A. (2002). The evolution and application of toxicity criteria. Semin. Radiat. Oncol..

[22] Trotti A. (2003). CTCAE v3.0: development of a comprehensive grading system for the adverse effects of cancer treatment. Semin. Radiat. Oncol..

[23] Basch E. (2006). Patient versus clinician symptom reporting using the national cancer Institute common terminology criteria for adverse events: results of a questionnaire-based study. Lancet Oncol..

[24] Basch E. (2009). Adverse symptom event reporting by patients vs clinicians: relationships with clinical outcomes. J. Natl. Cancer Inst..

[25] Feldman-Stewart B., Brundage M.D., Tong C. (2011). Information that affects patients' treatment choices for early stage prostate cancer: a review. Can. J. Urol..

[26] Eccles D.W., Arsal G. (2017). The think aloud method: what is it and how do I use it?. Qualitative Research in Sport, Exercise and Health.

[27] van den Haak M., De Jong M., Jan Schellens P. (2003). Retrospective vs. concurrent think-aloud protocols: testing the usability of an online library catalogue. Behav. Inf. Technol..

[28] Rezaee R. (2022). Development, usability and quality evaluation of the resilient mobile application for women with breast cancer. Health Sci. Rep..

[29] Gatto S.L., Tak S. (2008). Computer, internet, and email use among older adults: benefits and barriers. Educ. Gerontol..

[30] Tsetsi E., Rains S.A. (2017). Smartphone Internet access and use: extending the digital divide and usage gap. Mob. Media Commun..

[31] Aiyegbusi O.L. (2022). Key considerations to reduce or address respondent burden in patient-reported outcome (PRO) data collection. Nat. Commun..

[32] Snyder C. (2019). Making a picture worth a thousand numbers: recommendations for graphically displaying patient-reported outcomes data. Qual. Life Res..

[33] PROTEUS Consortium. 14/12/2022]; Available from: https://theproteusconsortium.org/proteus-trials/study-reporting/displaying-results/.

[34] Bennett A.V. (2016). Mode equivalence and acceptability of tablet computer-, interactive voice response system-, and paper-based administration of the U.S. National Cancer Institute's Patient-Reported Outcomes version of the Common Terminology Criteria for Adverse Events (PRO-CTCAE). Health Qual. Life Outcome.

